# Supplementary material for: Focused deterrence: A protocol for a realist multisite randomised controlled trial for evaluating a violence prevention intervention in the UK
Source: PLoS One. 2024 Mar 28;19(3):e0301023. doi: 10.1371/journal.pone.0301023 (PMC10977732; doi:10.1371/journal.pone.0301023)
Supplement: S1 File — The is the code to reproduce the power calculations. (PDF) [file pone.0301023.s001.pdf]

Power simulations

```
# FD ACF1 power calculations
# Simulation for negative binomial regression (count variable)

library(MASS)
library(tidyverse)
library(plyr)

# Set up and practice run

set.seed(422) # set seed for reproducible analyses

### Set these to change the sample size, effect size and data distributions ###

# Count variable
n <- 1700 # total sample size
alpha <- 0.05
d <- 0.75 # change in intervention group relative to control group
num_sims <- 100
mean <- 1 # mean of negative binomial outcome
var <- 1.5 # variance of outcome (>1 to be overdispersed )

###

###### Don't change anything below this line ######

id<-seq(1,n,1) # create ID variable beginning at 1
t1_outcome<-rnbinom(n, size= var, mu = mean) # create negative binomial baseline outcome
condition = sample(c(0,1), replace=TRUE, size=n) # randomly allocate each case to treat/ctrl
t1_risk = t1_outcome + rnorm(n, sd = sqrt(var) * 1.2) # generate 'risk' variable correlated with baseline
outcome
risk = cut(t1_risk, breaks = c(-Inf, 2.5, 5, Inf), labels = c("low", "medium", "high")) # turn t1_risk into integers

# Quick check on correlation between risk and baseline
risk_numeric <- as.numeric(risk)
cor(t1_outcome, risk_numeric)

##test that simulated data and model are functioning

# Simulate one data set
simdata<-as.data.frame(cbind(id, condition, t1_outcome, risk)) # create data frame

simdata <- simdata %>%
  mutate(t2_outcome = ifelse(condition==1, t1_outcome*d, t1_outcome))

# simdata <- simdata %>%
#   mutate(t2_outcome = round(ifelse(condition==1, t1_outcome*d, t1_outcome),0))

# Run model once
reg<-glm.nb(t2_outcome ~ condition + t1_outcome + risk, data = simdata) # regression equation

# Outputs of model
summary(reg) # get outputs of model
output<-summary(reg)$coefficients # get object containing model coefficients
coefs<-output[,1] # create object with coefficients
```

```
ps<-output[,4] # create object with p-values
results<-c(coefs, ps) # merge coefficients and p-values

names(results)<-c('Intercept_coef', 'condition_coef', 't1_outcome_coef', 'risk_coef', 'Intercept_p',
'condition_p', 't1_outcome_p', 'risk_p') # give useful names to results

results # output results to check

mean_control <- simdata %>%
  filter(condition==0) %>%
  summarise(mean = mean(t2_outcome))
mean_intervention<-simdata %>%
  filter(condition==1) %>%
  summarise(mean = mean(t2_outcome))
sd <- sd(simdata$t2_outcome)
cohen_d <- (mean_control - mean_intervention)/sd
cohen_d


## Create function that simulates data and runs model sims number of times

# generate a function 'regression_sim' that simulates the data, runs the regression model and stores estimates
and p-values

set.seed(123)
regression_sim <- function(simNum, n, b0, b1, b2, b3, p0, p1, p2, p3) {

  id<-seq(1,n,1) # create ID variable beginning at 1
  t1_outcome<-rnbinom(n, size= var, mu = mean) # create negative binomial baseline outcome
  condition = sample(c(0,1), replace=TRUE, size=n) # randomly allocate each case to treat/ctrl
  t1_risk = t1_outcome + rnorm(n, sd = sqrt(var) * 1.2) # generate 'risk' variable correlated with baseline
outcome
  risk = cut(t1_risk, breaks = c(-Inf, 2.5, 5, Inf), labels = c("low", "medium", "high")) # turn t1_risk into integers

  simdata<-as.data.frame(cbind(id, condition, t1_outcome, risk)) # create data frame
  simdata <- simdata %>%
    mutate(t2_outcome = round(ifelse(condition==1, t1_outcome*d, t1_outcome),0))

  # Run model once
  reg<-glm.nb(t2_outcome ~ condition + t1_outcome + risk, data = simdata) # regression equation

  # Outputs of model
  summary(reg) # get outputs of model
  output<-summary(reg)$coefficients # get object containing model coefficients
  coefs<-output[,1] # create object with coefficients
  ps<-output[,4] # create object with p-values
  results<-c(coefs, ps) # merge coefficients and p-values

  names(results)<-c('Intercept_coef', 'condition_coef', 't1_outcome_coef', 'risk_coef', 'Intercept_p',
'condition_p', 't1_outcome_p', 'risk_p') # give useful names to results

  return(results)

}

# regression_sim(1, n, b0=results[1], b1=results[2], b2=results[3], b3=results[4], p0 = results[5], p1 = results[6],
p2 = results[7], p3 = results[8]) # set parameters for one simulation
```

```
sims<-ldply(1:num_sims, regression_sim, n, b0=results[1], b1=results[2], b2=results[4], b3=results[5], p1 = results[6], p2 = results[7], p3 = results[8])  # create a dataframe called 'sims' that holds the results of the simulations

power<-sum(sims$condition_p<0.05)/num_sims # power is the proportion of p-values for 'condition' that are less than 0.05
power # calls 'power'

sims %>%
  ggplot(aes(x=condition_p)) +
  geom_histogram(binwidth=0.005, aes(fill = condition_p<0.05)) +
  xlab("Distribution of simulated p-values") +
  ylab('Frequency of p-values') +
  labs(title = paste0("Simulated power calculation",' n=',n, ' d=', d, ' simulations=', num_sims)) +
  annotate("text", x=0.75, y=200, label= power, size=10)

ggsave(paste0("Simulated power calculations", ' n=',n, ' d=', d, ".png"))
```
