# Supplementary material for: Focused deterrence: A protocol for a realist multisite randomised controlled trial for evaluating a violence prevention intervention in the UK
Source: PLoS One. 2024 Mar 28;19(3):e0301023. doi: 10.1371/journal.pone.0301023 (PMC10977732; doi:10.1371/journal.pone.0301023)
Supplement: Attachment — Submitted filename: Response to Reviewers.pdf [file pone.0301023.s002.pdf]

Response to Reviewers
14th February 2024

Dear Editor,

Subject: Revision of Manuscript titled "Focussed deterrence: A Protocol for a realist multisite randomised controlled trial for evaluating a violence prevention intervention in the UK"

I am writing to submit our revised manuscript for consideration for publication in PLOS ONE. We would like to thank the reviewers for their very helpful and constructive comments, which have contributed to an improved manuscript. For your consideration, we include two revised versions of the manuscript, one with track changes included and the other with track changes accepted.

We accept the comments and have revised the manuscript accordingly. Please see below a summary of our response to reviewer comments:

Reviewer #1:

Comment: Thank you to the authors for their dedication to the field and development of this very important work. This is a foundation of the implementation of a program, however, does lack much of the data needed for rationale in power and statistical analysis.

Response: Accepted and revised. Please see below for more on the above revisions.

Comment: In the introduction, I would prefer to see more epidemiology focus on actual UK data, as opposed to citing US data. For a program that is based in the UK, it is a challenge to cite this as a public health problem from the US surgeon general. Data from the UK, especially in types of violence would be imperative in laying the foundation in the introduction as to the need for the program in the UK. Policy differences are important to discuss and also raise enough disparities between communities that may impact your background in the justification of the use of the type of the program. This was not addressed here.

Response: Accepted and revised. The introduction and second paragraph have been revised to place more emphasis on UK strategy and approaches, and impact on policies. Please see the track changes on pages 5-6.

Comment: For the methods, you discuss the rarity of the violent events, but you do not give any number of events in the region. This would be helpful for understanding the expansiveness of the problem. For this study, even if you do not have any data, you could report out how many violent events occurred in the previous 5 years in each location. This would assist in learning if the sites selected would be able to meet the expected enrollment.

Response: Accepted and revised. We have provided pre-baseline data to support these assertions, which are included in the preregistration referred to in the manuscript.

Comment: This study states it is key on having adequate sample size. There are no sample size or power calculations listed, however this is stated several times. Please include these. You only include the sample size in the abstract, but not anywhere in your manuscript, along with the calculations. Please include these and explain rationale for the calculation. Overall, the explanations tend to not be clear. You are not explaining what you will be analyzing where, and your description of mixed methods evaluation is not clear as well. Maybe a table to supplement your planned analyses, or restructuring the section to have it grouped to better explain you plans.

Response: Accepted and revised. Further information regarding the sample size and power calculations is included in the track changes on pages 8-13. To improve on the explanation of the evaluation, further information has been added through the paper, specifically track changes on page 21.

Comment: Discussion: in your discussion was the first time you address sample size within the actual manuscript (outside of the abstract)- however having that data earlier would be important, as well as clarifying goal sample sizes for each site. You discuss that you plan to share on global stages however what are your anticipated outcomes and what barriers do you expect to encounter?

Response: Accepted and revised. This has been addressed throughout the paper.

Comment: Overall the general organization of the manuscript can be improved to allow better understanding of the study and planned statistical approach.

Response: Accepted and revised. This has been addressed throughout the paper.

Comment: There are no references included -- will need these with choices for sample size calculation, and in your introduction as well.

Response: Accepted and revised. Reference information has now been included.

Reviewer #2:

Comment: Summative Impact evaluation: What is the intervention date for these research questions? At randomization? The authors should clarify.

Response: Accepted and revised. Please see the track changes on page 9.

Comment: Outcome measures: Q2 and Q3 could use some clarification about what "PNC disposal outcome relevant to the evaluation" means. Could the authors provide an example?

Response: Accepted and revised. Please see the track changes on page 11 and 16.

As requested, here is the revised funding statement: This work was supported by The Youth Endowment Fund under grant number 3928041. There was no additional external funding received for this study. The funders had no role in study design, data collection and analysis, decision to publish, or preparation of the manuscript.

As previously stated, given its size and complexity, our study protocol necessitated a more comprehensive presentation to ensure clarity and thoroughness. The additional length is crucial for understanding the research and its implications. We hope the editorial team will consider our submission in light of the protocol's complexity, significance and uniqueness.

Our research has received ethical approval from the University of Hull Research Ethics Committee, and we have ensured the integrity and quality of our study. We believe our protocol and subsequent findings will be of significant interest to PLOS ONE readers, offering valuable insights into violence prevention strategies and their potential applicability in diverse contexts.

Thank you for considering our revised submission. We look forward to contributing to PLOS ONE and are available for any further information or clarifications you might require.

Kind regards,

Paul McFarlane for all authors
